# The Odontological Society of Great Britain

**Published:** 1891-04

**Authors:** 


AMERICAN JOURNAL

—OF—

DENTAL SCIENCE.

VOL. XXIV. BALTIMORE, APRIL, 1891. NO. 12.

ARTICLE I.

### THE ODONTOLOGICAL SOCIETY OF GREAT BRITAIN.

The Ordinary Monthly Meeting of the above Society was held on the 2nd March. The President, Mr. S. J. Hutchinson, M. R. C. S., L. D. S. Eng., in the chair, and a full attendance of members. The minutes of the previous meeting were read and confirmed.

Dr. A. W. W. Baker's (of Dublin) admission to membership was announced, and Mr. T. Maudsley Howkins, of Hull, was elected a member of the Society.

Mr. H. B. Gill, Upper Norwood, was proposed for membership.

Mr. Storer Bennett (on behalf of the Librarian) stated that two papers had been sent for the library of the Society, viz., the Calendar of the Pharmaceutical Society, and the October Number of the Transactions of the Odontological Society of Germany. He mentioned also (in his capacity as Curator) that two models had been presented to the Society by Mr. Bulkley Hughes, of Dumfries. One, an upper model showing two supernumerary teeth erupting in the palate. In this case it was stated by Mr. Hughes that a considerable amount of hæmorrhage attended the extraction of the teeth. The other model was taken for a boy aged fourteen, and showed a conical supernumerary tooth, which was also extracted. Mr. Storer Bennett, continuing, related the particulars of a case of alveolar abscess of three years standing, and remarked that it was exceedingly interesting and instructive. It was that of a root of an upper lateral incisor which was under treatment for four years, more or less. The case forcibly illustrated the disastrous consequences which may supervene from cleaning out a root canal with a drill which is not properly directed, owing to the risk of piercing the wall on one side or the other. In 1886 the patient, a young lady, then about fifteen years of age, had a cavity on the palatine surface of a right upper lateral incisor filled. About a year after a sinus formed and pointed opposite the apex of the tooth. The dentist whom she consulted then removed the filling, enlarged the root canal with a drill, and filled the canal with gutta percha. This made matters worse, for a second fistula formed and discharged through the gum below the first one. The patient therefore went to another dentist, who removed the gutta-percha, but without giving relief. She thereupon went to a third dentist, who very confidently expressed himself capable of treating the case. He, however, treated it on alternate days for six weeks without any signs of the discharge getting less. He then suggested that a London dentist should be consulted, which was done. The London dentist stated it as his opinion that the discharge was not in any way due to the abscess at the end of the root, but suggested that possibly there had been a perforation; a suggestion which an examination of the specimen would show to be correct. A short time after this the patient had occasion to see a surgeon about her throat, and he said its condition was not due to the teeth. The patient was now recommended to have the tooth removed, but after the unsuccessful attempts to give relief, extending over three or four years, she was not at all willing to consent to have the tooth extracted, but she ultimately did so, when it was discovered that the drill had been directed too far forward, and had pierced the anterior wall, while the plug of gutta percha with which the root had been filled had been driven through the foramen, and it was due to this that all the subsequent mischief had occurred. The specimen (handed round) spoke for itself, but Mr. Storer Bennett thought that in addition to the importance of not using a drill which would cut a channel for itself, but rather an augur drill, the case should impress upon them the necessity of not too readily assuming that a case is easy of treatment, especially when it has already been under the care of somebody else, who in all probability has given it thoughtful attention.

The President said that with regard to the casual communication of Mr. Storer Bennett, it would facilitate discussion if the specimen were handed round, and any remarks deferred until after the specimen had been seen. In the meanwhile he would call upon Mr. Ackery for a casual communication.

Mr. J. Ackery said that in bringing forward the two or three cases to which he was about to call attention, he was afraid he was doing so without being able to give any very distinct history. The previous history had not been well known, but the fact in itself gave the greatest scope for discussion as to the cause of the abnormalities.

In the first instance the model he wished to show was that of a lad aged fifteen or sixteen, in which the right lower first molar had become impacted, remaining on a lower level than the second molar. His object in bringing the case before the Society, was to get hints as to its probable cause. The puzzle was how a six year old molar could become impacted in this way. In his experience it was unique. The tooth was carious at its posterior aspect, and as the patient had not got his wisdom teeth, Mr. Ackery though it best to remove the misplaced tooth. This he did, and the wisdom tooth afterwards erupted very nearly in the position of the second molar. Another case quite inexplicable to his mind, was that which occurred in a member of his own famlly. The second upper molar had never come down to its true level. The first molar was tilted forward. There had never been any irregularity in the lower teeth to account for the position of the upper, and it had occurred to him whether there was gemination at the roots.

The third case was that of a medical man, in whose mouth the right upper second bicuspid was only partially erupted, high above the alveolar border in the sulcus. The tooth showing signs of caries, and being quite useless, Mr. Ackery thought it better to extract, before the presenting portion became broken down. On removal the root was found to be of less than half the normal length, and quite flat and even. (The tooth was shown.) Mr. Ackery said the appearance of the end of root was not like an ordinary absorbed surface, it was certainly not fractured, but might possibly be due to some interference with complete calcification. The specimen he hoped to have microscoped, and shown at some subsequent meeting.

The fourth case was simply an upper model, showing both temporary and permanent canine, simultaneously present on either side of the mouth, and in the true arch, but on the right side the bicuspids were rotated, so that their long axes were antroposteror, and they were lying side by side, one being lingual and the other labial.

Mr. David Hepburn, being called upon by the President, said that he did not propose to occupy the time of the Society for more than a few moments, but very often in ordinary practice some contrivance suggested itself which proved to be a convenience, and which, when used, after a time became indispensable. The dressing stand (shown) was devised so as to form a convenient receptacle for small quantities of drugs for daily use; it was circular and pyramidal in form, and revolved. It consisted of three tiers, reduced to the smallest possible dimensions consistent with utility. The lowest tier was provided with a number of small boxes for amadou, gutta percha pellets, bibulous paper pellets, and so forth. The second tier contained receptacles for bottles without necks, and with covers on hinges. The third tier was for the spirit lamp. Mr. Hepburn suggested that for the purpose of readily recognizing the whereabouts of the particular drug desired to be used, the bottles might be of different colors; thus pink might be associated with carbolic acid, and green with arsenic. This plan might also be extended to the bibulous paper pellets. He had found the arrangement of considerable use himself, and therefore ventured to bring it before the Society.

The President mentioned that Dr. Scanes Spicer had been good enough to bring two patients for them to see, and as it would be necessary to see them in a darkened room, perhaps it would be convenient to do so at the close of the meeting. Mr. Ashley Barrett had also brought a patient, showing a case of double dislocation of the jaw, and he would call upon Mr. Barrett for his communication in connection with it.

Mr. Ashley Barrett said that the patient presented herself at the out-department of the London Hospital about three months ago, and complained of almost complete inability to open her jaws. There was considerable pain on the left side of the mouth in the neighborhood of the wisdom teeth. Mr. Barrett opened the mouth, under chloroform, with a screw opener, finding it necessary to use only a moderate amount of force. He extracted the molar—a certain amount of inflammation was going on round it, and in the neighborhood of the masseter muscle. In January the patient come again to the hospital, and said that every time she opened her mouth the jaw clicked, and on examination, apparently the condyle of the inferior maxilla slid over the articulatory prominence, and when she closed her mouth again the dislocation on both sides was reduced. She said it all but entirely prevented her eating. There was a good deal of pain, and the clicking noise produced constant headaches. The points upon which Mr. Barrett would be glad to have the opinions of members were, first, the cause of the dislocation, and secondly, the best mode of giving relief. With regard to the cause, it seemed to him to be a somewhat obscure matter. On consideration Mr. Barrett thought three possible conditions might have caused it. The patient was a young woman of loose fibre, aged 24, and complained of clicking in every joint. The right shoulder joint dislocated, and the left knee. It occurred to him that possibly she might be affected with rheumatoid arthritis, which sometimes affects the jaw; there might be, accompanied with this, a growth of bone about the glenoid cavity, which might be the cause of the pushing forward of the lower jaw. Mr. Barrett was not quite clear in his own mind whether complete dislocation existed. The second suggestion was that possibly, though he was not conscious of it, he might, in opening the mouth with the mouth opener, have done some violence to the capsular ligaments. The third possible cause which occurred to him was that possibly the capsular ligaments might have wasted and become very lax, the external pterygoids might also act in an inco-ordinate manner, and they might draw the condyles forward. Mr. Barrett could not account for the condition, except on one of those three hypotheses. As to relief, it occurred to him that this slipping of the jaw might be prevented by mechanical means, and for this purpose he had constructed a head-cap with bands which connected it with a chincap, made of vulcanite, so that a constant traction might be exercised on the jaw, and he was hopeful that by contiued action, the condition might be improved. Further, he might mention that the patient had no front teeth, a circumstance which he proposed to utilize by making a strong metal plate, with rather long upper incisors, which he hoped would prevent the jaw slipping forward.

The President remarked that they seemed now to have a considerable amount of matter to discuss. Perhaps it would be better if any members had remarks to make on Mr. Storer Bennett's case, that they should be made now.

Mr. W. H. Coffin wished to say that about a year ago a lady came to him under almost similar conditions to those described by Mr. Bennett, the only difference being that the lateral incisor root carried a pivot and an Ash's tube tooth on a gold pin. Every effort to alleviate the discharge failed, and Mr. Coffin extracted the root with the pivot, and found as he had expected that there had been a perforation, in almost the same position as in Mr. Bennett's case. The gold pivot protruded about 1-32 inch. After removing the pivot and making the root smooth on the outside, he replanted it and it became perfectly firm. Mr. Coffin afterwards mounted a Logan crown on the root and the case was doing perfectly well. He mentioned this case because it would encourage them to extract and replant a similar tooth.

Mr. F. J. Bennett commenting upon Mr. Barrett's case, said that it scarcely would appear to be a true case of dislocation of the lower jaw. It seemed to him to be more a gliding forward movement on the condyle and was probably due to the relaxation of the ligaments and increased by constant repetition. Probably if such a splint as Mr. Barrett had suggested were rigorously applied for a few months, it would restore the jaw to a proper movement. In such cases he believed the explanation was that the condyle was unusually flat, and the glenoid cavity unusually shallow. In such circumstances it was not rare to find a little forward movement of the jaw, but not a true dislocation. With regard to Mr. Ackery's case, from his description it reminded Mr. Bennett of a case he had seen in private practice. The first lower molar was impacted between the second lower molar and bicuspid in front; this was exposed and of such a curious shape that Mr. Bennett thought it was a denticle, but he found on extracting it that it was an illformed tooth with a conical fang. Mr. Bennett believed that it had been pointed out by Tomes and others that the position was largely due to the growth of the fang, and therefore he concluded in his own case that in consequence of the fang being much dwarfed and conical, it had been overtaken by the second molar and bicuspid, and once being behind in the race had been held out of position by the other teeth.

Mr. F. Newland-Pedley expressed his agreement with Mr. F. J. Bennett as to the nature of Mr. Barrett's case. He did not for a moment think it was a case of perfect dislocation. He had the pleasure to exhibit a model of the mouth of a man, aged twenty-four, who inherited syphilis. When about six years of age he got necrosis in the palate, and about four years afterward he erupted a tooth.

Mr. W. H. Coffin then exhibited a paper disc with a peculiar central perforation, the invention of Dr. J. A. Kimball, of New York, consisting of a combined slit and hole allowing the flat head of a screw mandril to be passed through bodily, without the necessity of completely removing the screw from the mandril.

Dr. Job Collins then read a paper on

"ASSOCIATED AND RELATED OCULAR AND DENTAL DISEASES."

Bain had observed "Nowhere more than in medicine may laws of causation be defeated; there is rarely such a thing as a simple cause yielding a simple effect." Power had indeed stated his belief in a causal connection between phlyctenular ophthalmia and carious teeth, but on another page he recognized the ambiguity of such a conclusion, owing to the extreme frequency with which dental affections present themselves. Statistics then, said Dr. Collins, seem of little avail, and a deductive method of proof and judicious empiricism seemed the only resort. On the other hand, Hutchinson from an examination of 102 cases of interstitial keratitis was able to arrive at the induction that the disease was due to inherited syphilis, and was very frequently accompanied with a characteristic physiognomy and definite irregularity. The same author's demonstration of the remarkable concomitance of laminar cataract, defective enamel, especially of the first molars, and Arlt's connection of the former with infantile convulsions, were not less suggestive of some underlying relation between ocular, dental and general aberration. Bearing in mind the destination of the whole of the 2nd, 3rd, 4th, 6th and parts of the 5th and 7th cranial nerves, together with the sympathetic to the eye and orbital contents, eyelids and muscular appurtenances, and the associated rich innervation of the teeth by the other two large sensory divisions of the great trigeminal nerve, it was impossible to fail to appreciate the opportunity of inter-communication afforded. Accepting the truths which physiology teaches of relaxation, transference, radiation and so-called "sympathy," there seemed an *a priori* probability that in certain circumstances conditions must arise in which afferent impulses ascending the dental nerves should manifest divers sensory and motor phenomena, in the complex system composing the organ of vision. There were affinities developmental, histological and general between the eyes and teeth which must not be lost sight of when taking a broad view of the two factors whose interaction was to be investigated developmentally; an analogy by no means fanciful might be found in the fact that with the eyes, as with the teeth, an epiblastic invagination co-operated with a vascular mesoblastic intrusion in the genesis of the organ. A liability to sympathetic tendencies and lesions might thus be laid at the outset. The connecting link of non-vascularity of the dentine and enamel and cornea and lens was worthy of remark. The correspondence of the two sets of phenomena might be less true in point of time than in essence, and in their later life histories there was little of analogy between the cyclic changes of the two dentitions, and the age changes which eventuate with the eyes. Prefacing a citation of cases with the remark that the indirect class, especially such as were recorded before the days of the ophthalmoscope, and before Donders demonstrated the pathology of concomitant squint—*i. e.*, before the sixties—were of little value. Dr. Collins referred to the case quoted by Sir Wm. Lawrence from Galenzanski, spoken of by Mr. Power as the most brilliant and complete on record of reflex amaurosis from irritation of dental branches of the fifth. It was the case of a man aged thirty, in whom a splinter of wood perforated the fang of the first left upper molar which was carious; swelling and pain of the cheek on that side occurred, and later on mydriasis and blindness, which was said to have been complete; this lasted a twelve month, and when the cause was detected by Galenzanski and the tooth with the splinter removed, restoration of sight quickly ensued. The effect of an orbital cellulitis directly upon the optic nerve and the striking and rapid improvement of sight after evacuation of inflammatory effusion, was well shown in a case recorded by Mr. Critchett. Dr. Collins stated that cases of definite ocular disease for which a dental cause could be satisfactorily established were very decidedly rare. He had searched the ten extant volumes of the Ophthalmological Society in vain for a clear case of the kind classed in his second group. He found a case of fatal orbital cellulitis resulting from periodontitis and necrosis of jaw, and one of associated neuralgia or anæsthesia of the fifth, with ocular troubles; but no reflex amaurosis or phlyctenular ophthalmia, or glaucoma or ophthalmoplegia, or failure of accommodation attributed with any assurance of proof to dental lesion. Mr. Power's comprehensive paper, which laid under contribution most of the available literature on the subject up to that date, yet only contained two detailed cases which had come within Mr. Power's own experience. One was a young woman with neuro-paralytic ulcer of the cornea; she had corneal anæsthesia and ulceration with hypopyon, and though there was some improvements after taking out some teeth, yet the eye subsequently had to be abscised. The other case was one of double glaucoma in which a cystoid cicatrix followed each iridectomy, and both eyes were lost. She had had toothache, and an abscess was always found to exist at the root. Mr. Power did not seem either to suspect or assert dental cause in this case, nor did more extended knowledge of the pathology of glaucoma since acquired, suggest it as likely. Indeed Mr. Priestley Smith by measuring the tension of the eye in cases of toothache at the Dental Hospital, found no reason for thinking that toothache affected ocular tension, and though his number of observations were but few, yet there seemed good ground for his conclusion, "That the part played by ordinary forms of facial neuralgia in glaucoma is not of primary importance."

In the discussion which followed Mr. Power's paper, Dr. Collins was only able to find records of eight cases of oculo-dental lesion contributed by the sixteen speakers. Mr. Smith Turner related a case of amaurosis from antral abscess. Mr. Charters White mentioned a case of keratitis with a carious bicuspid. Mr. Coleman brought forward a case of amaurosis from pivoting a left upper central, and added that conjunctivitis was the commonest ophthalmic trouble resulting from dental irritation. The amaurosis case seemed to be the same as elsewhere referred to, and it was stated by Mr. F. J. Bennett on the authority of Mr. Lawson, that the ocular trouble was iritis, and in no way due to reflex dental irritation. Mr. Spence Bate related a case of neuralgia in the eye relieved by the removal of carious upper molars, and three or four other speakers quoted cases in which ocular and dental troubles were supposed to be more or less related to each other. Dental surgeons were evidently on the alert for cases of the kind to which Dr. Collins was referring, yet the records of the transactions of the Odontological Society were few and far between. Mr. Henry Sewill had related and reproduced in his "Dental Surgery" a most interesting case of spasm of the obicularis, neuralgia and cataract, apparently due to tender, carious and much neglected teeth on the same side. Appropriate treatment of the teeth cured the spasm and neuralgia, but of course not the cataract. Having quoted other cases, both from his own case-book and other sources, Dr. Collins proceeded to say that he had never seen a case of glaucoma, acute or chronic, which appeared to be in any way caused by diseases of the teeth; the neuralgia of the head and cheek was common enough in such cases. He remembered a case of intense supra-orbital neuralgia, which was puzzling; later, however, the neuralgia gave way to total anæsthesia over the area of distribution of the supra-orbital nerve. On deep pressure, a lump was felt, evidently involving the supra orbital nerve trunk, this was removed, was as large as an almond, and was composed of granulation tissue; there was a history of syphilis, and it was possibly a node. Dr. Collins had paid a good deal of attention to failure of the accommodation, and, notwithstanding the statements of H. Schmidt, he much doubted whether dental neuralgia had any other effect in this direction than that of general depression, and consequently, as with so many other deflecting causes, as blood losses, the puerperal state, hyper-lactation, convalescence from exhausting fevers, in a measure restrict the range of accommodation. In sixteen persons suffering from toothache tested by Dr. Priestly Smith, fifteen had no anomaly of accommodation. Nor did Dr. Collins think paralytic affections of the external or internal muscles of the eye or lids were often, if ever, traceable to dental disease. A reflex paralysis presented some physiological difficulties, and a reference to "inhibition," which seemed to imply some normally cxerted motor influence, did not elucidate the matter to his mind. He felt obliged to confess to scepticism as to the satisfactory demonstration of cases of reflex amaurosis arising from irritation of the dental nerves, apart from direct extension of inflammation through the maxilla. Such cases became more frequent in literature as the student receded into pre-ophthalmoscopic and less critical times, and became rarer and seemed to disappear when they were most vigilantly enquired for. The deep origins of the optic nerve, though not precisely determined, would appear to be far removed from the fifth, and the path of such reflex amaurosis proportionately circuitous and improbable. Of associated ocular and dental lesions, such as keratitis and notched teeth, and lanular cataract and "rachite" teeth, every ophthalmic surgeon had abundant instances at hand. But it was curious to notice how often many who should know better appeared to confound the normal triple denticles which the permanent incisors exhibit normally when first cut, and which often remain some years before they are ground down with Hutchinsonian notched teeth of hereditary syphilis. It is, Dr. Collins submitted, by the defective enamel of the central denticle causing this to be broken away that the notched appearance results, and he exhibited a photograph which served to illustrate this process.

The President said, with reference to the paper which Dr. Collins had just read, he was quite sure he was only expressing the unanimous feeling in thanking him for his extremely valuable contribution. If this iconoclastic paper had smashed their idols, they must accept their fate and not so hastily jump at conclusions which some appeared to have done, he (the President), amongst the number.

Mr. F. J. Bennett said, with reference to Dr. Collins's allusion to the case which occurred while he (Mr. Bennett) was a student at the hospital, and which caused some interest at the time as to the doubt expressed whether or not it was a true case of reflex amaurosis, the case wss one in which the patient having a lateral tooth pivoted, suffered from iritis. It was due to the pivoted tooth that she was first taken to the hospital, and seen by Mr. Lawson himself. Mr. Lawson expressed himself in most decided terms as to the iritis being in no way of dental origin. Mr. Coleman was of a different opinion, and had expressed his opinion on more than one occasion. Mr. Bennett had always felt it his duty to put this fact on record, whenever this case was put forward as an example of a connection between amaurosis and dental trouble. It should be remembered that this case was seen by no other ophthalmic surgeon, a fact which he thought should be noted; he believed Mr. Tomes had expressed himself in a guarded way, and he classed it among doubtful cases.

Mr. Albert mentioned, as illustrating the difficulty of tracing the relation between cause and effect, the case of a little child of his acquaintance, aged about seven or ten, suffering from ulceration of the cornea. She was taken to one of the first oculists in London, and he treated her in the orthodox way. After three or four months, as the ulceration did not clear up, she consulted Mr. Power, who attributed the lesion to a temporary molar; this was extracted and the ulceration disappeared.

Mr. C S. Tomes wished to say one word about a case Dr. Collins had alluded to, seen by Mr. Critchett. A case of repeated attacks of cellulitis, coincident with which there existed a peculiar condition of the mouth. It was a remarkable case; the patient was very bad indeed, and seemed almost to be dying. There was never any suppuration, only serum. The teeth were very stunted from a girl, and there were very few permanent teeth. Those that she had were very stunted, pointed, peg-like sort of teeth with hardly any enamel, and they were erupted to a very small extent only. But on no occasion upon which he saw the patient, was there any inflammation about the necks of the teeth, though he did not see her quite in the first instance. Some of the teeth which were removed were sound. The recurrence of the cellulitis, and dental pecularities, did not suggest cause and effect, but rather concomitant conditions. He could not, in his own mind, connect the condition as cause and effect, nor could he think of any case like it. But there they were, the very stunted teeth, cut long after the normal period, and at long intervals. It did occur that cellulitis was coincident with the cutting of the teeth. But there was no abscess or inflammatory condition.

Mr. W. A. Hunt, Yeovil, remembered being present when Mr. Power read his most interesting paper. It was a paper which produced a suspicion that there was a connection between conditions of the eye and of the teeth. He thought

Dr. Collins' sceptical paper would induce the dental profession to examine very carefully the evidence brought forward in support of any supposed connection between diseases of the eye and teeth. Dr. Collins's paper had been read in post-ophthalmoscopic, not pre-ophthalmoscopic times, and Mr. Hunt thought that there existed now more than ever a very strong suspicion of the connection between certain dental and ophthalmic conditions. They might not have all the evidence that Dr. Collins required, but as he had said eight years ago, he hoped that careful notes would be taken of any cases affording grounds for supposing a relation of the one and the other, so that the details might give something definite, and no mere hypothesis to work upon. He believed, and he was speaking from his own experience, that there *was* a connection between certain diseases of the eye and teeth. When surgeons were found removing one eye lest the other might be affected; when constitutional and local disturbances were shown to be related to each other, he could not help thinking that there might be a connection between functional disturbance of the eye and diseases of the teeth.

Mr. F. J. Colyer wished to mention a case of slight corneal ulcer treated by Mr. Power of Westminster Ophthalmic Hospital, without success. In the temporary molar there was a fig pip which was pressing on the exposed nerve. On removal of the tooth the corneal lesion got well.

Mr. Morton Smale thought that dentists were a little inclined sometimes to jump at hasty conclusions, and that they should therefore be very much indebted to Dr. Collins for breaking up some of their idols. After Mr. Power's paper, Mr. Smale began to think he had been very neglectful in failing to observe related conditions between the eye and teeth. Shortly after, a colleague at Westminster Hospital sent him a case of glaucoma in which there were some decayed stumps on the side of the glaucoma, of which he thought they might probably be the cause. After Mr. Power's paper, Mr. Smale thought so too, and he accordingly removed the stumps. Things went on very nicely indeed for about a year, when he saw his patient again, and congratulated him on the cure of the glaucoma; he had hardly done so when the glaucoma recurred. This case had hardly left him, when an almost similar one was sent him by a fellow student, who thought the glaucoma might be due to the decayed teeth, but in spite of the removal of these teeth by Mr. Smale, and putting the mouth in good order, the glaucoma continued, and the patient had finally to be operated upon.

Mr. Paterson after expressing the pleasure with which he had listened to the paper, notwithstanding it had bowled over their idols, alluded to the obvious nervous connection between the eye and teeth, in view of which, he said, irritation of either might be expected to affect the other. He mentioned the case of a superior canine the pulp of which was dead, and the tooth so much decayed as to have little more than the stump left, which had been subjected to abscesses; this was attended with severe lachrymation on the same side, but the lachrymation disappeared on the removal of the stump. Another case was that of a supernumerary bicuspid-like tooth, carious and abscessed, also attended with lachrymation, which vanished on extraction of the tooth. Mr. Paterson, in conclusion, urged the importance of ophthalmists co-operating with dentists in recording and comparing the treatment and result of cases.

Dr. Collins briefly replied.

Dr. Scanes Spicer's two patients were then examined in a darkened room. One a male and the other a female, were each suffering from empyema of the antrum on one side of the face only, the other side being healthy. A small incandescent electric bulb having a reflector which projected the light upwards was placed in the mouth, which was then closed. The external transluence of the healthy side of the antral region was very decided, more particularly in the neighborhood of the canthus, but on the side where the infiltration existed there was no illumination.

The usual votes of thanks concluded the meeting.—*Dental Record.*

